# Pulpit and Pew

**Published:** 1869-04

**Authors:** 


### PULPIT AND PEW.

"A BEGGARLY account of empty boxes" is what we read and hear of all over the land in reference to our churches. This is not because the pulpit is not ably filled. There are men of ability, piety, learning and culture in New York city, who can scarcely command three hundred hearers on a beautiful Sabbath morning in "The May." Yet, when the "ball is up," and there is ice in the Central Park, there will be ten thousand skaters on the lakes, at the expense of a two miles walk or a five miles ride, although old Boreas blows his boldest blasts; it is because the pulpit is less attractive than the pond. Why less attractive? How less attractive? Who shall answer these questions? What is the most truthful reply? We have the fact; but we want the cause of the fact, because we doctors can do nothing curative until the cause of the disease is ascertained and then removed. There are many reasons why the people do not go to church more than they do, and different persons will give different reasons: the wickedness of the human heart; an almost unconquerable indifference to the great concerns of an eternal world; the strife for bread; the absorbing nature of worldly care, of business engagements, are all causes of the evil of empty churches; but why do not these avail to prevent New York city paying fifteen thousand dollars every night to be amused at theatres and ball rooms, and low dance houses? We have it in our mind to name only one cause why people do not go to church more than they do in this latitude of New York city; readers elsewhere must judge for themselves to what extent this is an operating cause in their own localities. In matters of this sort, there is nothing like making a bold charge, plain and explicit. The people would go to church more than they do, but—

THEY ARE NOT WELCOME!!!

as we shall prove conclusively enough before this article is concluded. It is not meant that the clergy would not welcome crowded houses with outstretched arms and joyful, thankful, humble hearts; and there is perhaps not a member of the church to be found who would not feel gratified to have his place of worship filled every Sunday to its utmost capacity, except that he would rather prefer his own pew should hold only his own family. Here is the rub; this is where the shoe pinches. Each man wants every pew crowded, except his own. He feels somehow or other that he has a right to it; his money pays for it, and to have it occupied by strangers is irritating. That is all true. But you and Paul differ a little in your practical theology; he bought his "Porter House," and his "Tender Loin," and his "Spring Chickens," and his "Canvas Backs," and paid for them, but in thinking the matter over, he drew back exclaiming, "if meat cause my brother to offend," I will never eat another mouthful as long as I live. These were not his exact words. We are in "The Lady's Chamber," and our concordance is down at 176 Broadway, standing side by side with the American "Dispensatory," for we like to be literally exact when we give a patient a recipe; but the meaning of the stern, sturdy and sterling old "Roman Citizen" was, that for the love of God, for the love of souls, he was willing to submit to self-sacrifices and personal inconvenience, if thereby he might save some.

In writing for our Journal, we have always had a special desire to speak from "the card," to fortify our positions from observed incidents coming under our own eye. And now for a very interesting story of fact about being

INVITED OUT, not to a duel, nor a horse race, nor a dinner party, but in this wise. Last summer, in the beautiful leafy June, we had a desire to hear a Fifth Avenue preacher, and as our own church was always crowded to repletion we thought our seat would be filled by some stranger and we would not be missed; knowing a friend who had a pew, we obtained permission to occupy a seat in it. We were greatly edified. At the conclusion of the services, it was stated that the regular pastor would occupy the pulpit on the next Sabbath day for the last time during the summer, but that the house would be opened all the season, the services of another having been secured; the pastor expressed the hope that the members of another church, which would be closed for repairs, would be welcomed to worship with them; also all strangers who might chance to drop in. Accordingly we were on hand bright and early next Sabbath morning with some ladies. There were about a dozen persons in the building, and not seeing any one to show a seat, nor liking to stand an indefinite time, we thought that under all the circumstances of the case, there was to be a "free fight," and we might very properly take a seat where it suited us. And so we did; going up the middle aisle, and occupying a pew very near the pulpit. We acknowledge a weakness in that direction. When we go to church, we like to get close to the speaker so that we may hear every word, and not have our satisfaction lessened by a painful effort at listening; for it often happens that clergymen fail to enunciate their words distinctly, and you have to guess at about half they say; besides, it does not look well to have people crowd the doors of our churches, and leave the front seats vacant; it seems as if they were afraid of being "hooked in" in some way; as if they wanted to have a chance to break and run, if need be. Then again, we have often noticed that at all public lectures the most coveted seats are near the speaker's stand; they are the "reserved seats," and the knowing ones are willing to pay a higher price for them; all know that a minister preaches with greater ease, with greater satisfaction to himself and others, with greater unction, if his hearers are gathered at his feet.

Scarcely had we comfortably seated ourselves, when we were very courteously and yet hesitatingly asked by the sexton,

"Are you friends of Mr. Blank?"

"No, sir."

"Mr. Blank's family always attends church, would you have any objection to take another seat?"

"Not the slightest."

So we gathered up ourself and the ladies and were very kindly taken to another pew; we waited with some curiosity to see if Mr. Blank and his family would come that day. We concluded that he was one of the big men of the church; that his wife was a duchess, proud and portly, and that in the train there would be several daughters, grown and ungrown, and altogether a sight worth seeing. But we soon became so absorbed in another sight that Mr. Blank and his wife and his fair daughters were totally forgotten, and we really at this writing do not know whether they came that day or not; and then, again, the sermon came with such "convincing power and might," from one whose "praise is in all the churches," that we went away "fed" and glad.

This is the third time we have been invited out of pews in unequivocal terms in the dog days, when there was pew after pew, before and behind, up stairs and down stairs, right and left, without a solitary occupant; every time in a Presbyterian church, every time in mid summer, and every time after a public invitation from the pulpit, and twice from a special advertisement in the newspapers.

It cannot be supposed that the Presbyterian are less hospitable than other pewed churches, nor that it is a mere coincidence that we have been invited out of pews three times, in plain unmistakable words; the fair presumption is that it is the same in all places of public worship, where the pews are rented, and that thousands have been served in the same way but have not taken the trouble to make it known in a public manner. A lady friend informed us, that becoming dissatisfied with her own minister, she thought she would for one day go quietly to a Fourth Avenue Presbyterian church, and as it was not far from her own house, in Fifth Avenue, she did not order her carriage, but walked to the church, and that from the reputation of the minister, she went with a confidence that she "would be fed." Her feelings were so wounded in connection with obtaining a seat, she felt as though she would never go to church again except to her own pew.

"The owner wants his pew," said a Mercer street sexton one day, while there were entire slips without a single occupant within a few feet. Our experiences have been so dis couraging in church going, that now, we seldom, except when our own is closed for repairs, go anywhere, although there is a whole column under the head of "Religious Notices," in the Saturday papers, giving a "cordial" invitation to the public to attend. In reading them over we cannot avoid the feeling that it is all a sham, except in those cases where some Infidel, Atheist or other "Crazy Jane" is to hold forth. We have seen with great satisfaction that the Broadway Tabernacle is "free" every Sunday night, and we have wanted to go there to see if it were really so, but we have such a perfect dread of the stereotype fatality which seems to follow us, that we have not been able, as yet, to screw our courage up to the point to "come and see;" and the effect has been to drive us to that menagerie of wild beasts, in the estimation of many good men and women, the famous

St. Albans in Forty Seventh street, three blocks east of Fifth Avenue; there we feel at home; there is no nervous looking round at every footstep or dress rustle behind you, in fear that "the owner wants his pew;" there is a perfect security against all interruption; there is no half an hour of uneasy waiting at the door while the one sexton is tolling the bell, at the very time he is most needed to show strangers a seat.

We have heard several sermons in the St. Albans, and have never heard one that any pious Protestant or Roman Catholic could reasonably object to; they were plain, practical, earnest sermons, delivered without affectation, with becoming simplicity and apparent sincerity. We saw a number of things there which were entirely new to us, but we went to hear the preaching; "it was good," and we came away gratified and improved, intending to go again when our church is "closed for repairs," feeling assured that we need not wait a moment for a seat, and that when we reach it, we are secure from interruption until the close of the service. We hope that the day is not very distant when all churches will follow the example of St. Albans in several other practical respects, which were advocated in this Journal some ten or a dozen years ago. One of these was to have large churches, plain, and so arranged that each pew should be free and hold two or three persons at most, without doors, to avoid having three seats appropriated to one person, and his sitting at the entrance of the pew, closing the door and thus keeping others out, as is the very common custom now.

There are but three rules to be observed at St. Albans, all of which will commend themselves to the common sense and good judgment of every person of reflection.

1st. Take a seat, the further one unoccupied from the entrance, thus leaving the others to after comers.

2d. Remain until the services are over.

3d. To contribute what you feel free to do, as the expenses are to be paid by the liberality of the worshippers, there being no other source of support.

Hence, in going to worship at St. Albans you feel at home. "You pay your money and you take your choice;" in the pew rented churches, the plate is pretty certain to come round, when you have to pay your money, but have no choice at all.

We know there are difficulties experienced in supporting churches in large cities, without hiring out the pews, and it is a great convenience to be able to have one's family in the same pew; besides, a man beyond all question, has an exclusive legal right to the seat for which his money has paid; but then don't invite people to attend through the public papers; it would be more frank, honest and satisfactory to have a banner floating from the outer-wall with this inscription:

"STRANGERS AND POOR PEOPLE ARE NOT WANTED HERE."

But we don't want to be confined to St. Albans; we might become "heretical" in our old age, and it is not wise to go into the way of temptation; we have found out certain churches in "The Avenue," that have "mourner's benches," hard seats, without books, cushions, or anything else—we have of late occupied them, not with the intention of "crowding the mourners," but going with the conviction that there would be no danger of finding any mourners there; for all the congregation are rich and have nothing to mourn about.

Strangers who happen to be in New York on Sundays, will be sure to find a welcome to a free seat in the Episcopal church in Fourteenth street, east of Broadway, opposite the Academy of Music, by the liberality of some generous men. The same denomination have a free church in Fifth Avenue, east side Forty-fifth street, built by the large-heartedness of the Rector himself, Dr. Howland, a son of the honorable house of the olden time of "Howland and Aspinwall;" it is called "The Church of the Heavenly Rest." Is this so?

All honor also be to the warm-hearted disciples of good John Wesley, whose gospel has hitherto been as free as air, and the latch-strings of whose churches have never been pulled in. After all, the difficulty is not insuperable. Spurgeon's pews are rented; so are Beecher's; so are Hall's, on Fifth Avenue, corner of Nineteenth street, and yet they are always crowded. In Spurgeon's church, said to hold seven thousand persons, we did not see one vacant seat. The plan of Mr. Beecher is to have the doors closed to a certain moment against all strangers; after that, any unoccupied seat belongs to the public, and a number of gentlemen are deputized to fill them. At Spurgeon's, as soon as the carriage stopped at the curb, a policeman opened the door, and piloted us and the ladies across the sidewalk, across the area and up to the door of the church.

"Seats for five," said he to a gentleman standing at the door, with paper and pen in hand. "Seats for four in No. 520," said he to another person near by, "but by no possibility more than four," and thus, without the need of any ticket, seats were quickly and quietly found for the whole party; the simple arrangement having been that each pew-holder, in going in, or before, gave notice how many seats would be vacant in his pew that morning. We were greatly pleased with the simplicity and perfection of the arrangement; the more so, as in other years, in the good Dr. Alexander's time, we often made it a point to tell the sexton, on going in, how many seats would be unoccupied. It is earnestly hoped that every pewed church in New York will adopt this method promptly and as a permanency.

THIS IS NOT ALL.

A member of the church must not only give a cordial welcome to the stranger and the poor that is within his gates; that is but the negative of his duty. Instead of merely giving them a cordial welcome when they do come, you must go farther; invite them to come; go to their houses, tell them they will be welcome and persuade them to come; "compel them to come in" by the logic of reason, expostulation and love. That noble institution, the Young Men's Christian Association of one of our churches, visited six hundred boarding houses in a certain district, in every one of which there were more or less persons who did not go to church anywhere on Sundays.

In an admirable publication of Joseph M. Wilson, of Philadelphia, Pa.,—a statistical record of many church matters—it appears that there are enough houses of worship in the United States to accommodate with a seat every person over fifteen years of age. Many of the religious sects are subscribing large sums of money to build churches. Those noble-hearted Methodists of St. Paul's church, Fourth Avenue, New York, the Harpers, the Drews, and others like them, gave a hundred thousand dollars in a single day for the purpose of affording more room for those who wanted to hear the gospel preached. While, then, the men who have money are spending it with a large liberality in building more churches, how would it do for those who have no money but have a heart for God and humanity to make personal effort to have every house of worship crowded every Sunday, morning, noon and night. What a melancholy waste it is to have sermons uttered in New York every Sunday to three or four hundred people, which ought to be heard by as many thousands; sermons big with thought, convincing in their reasoning, powerful in their illustrations, earnest in their delivery, and deep in feeling and piety.

Protestantism is only another name for that temperance and righteousness of which Paul spoke in the presence of royalty, requiring "diligence in business," and denouncing him who "provideth not for his own household." To make Protestantism a success, then, is to make all men temperate, industrious, and good providers for their families; and who can not see that if these things were universally done, that human life and health and happiness would be promoted a hundred fold over the face of the whole world.

What then is required, after all, to make Protestantism a success in the highest sense of the term. Can it be brought to the mind in a single short sentence, easily understood and easily remembered? Fill our churches with hearers of the preached word. Bring the people to the house of God, for it is his ordained means of salvation. "The common people heard him gladly," is as true to-day in New York and in London as it was in Judea two thousand years ago. There is a certain attractiveness in the exhibition of "revealed truth" in all ages and among all people, only let there be no hindrance to the hearing. Let the rich build churches and let the poor fill them by their own presence, and by inducing others to come, and other Whitefields, and Halls, and Tyngs, and Spurgeons, and Wesleys will be developed for the occasion, for it cannot be doubted that there are many such in embryo to be brought out by the sustaining power of the church, and the miraculous stimulus of listening multitudes, and then, as was never before in all the world's history, will all see

PROTESTANTISM A SUCCESS.

